# A rapid review investigating the potential impact of a pandemic on the mental health of young people aged 12–25 years

**DOI:** 10.1017/ipm.2020.106

**Published:** 2020-09-11

**Authors:** A. O’Reilly, M. Tibbs, A. Booth, E. Doyle, B. McKeague, J. Moore

**Affiliations:** 1 Jigsaw – The National Centre for Youth Mental Health, Dublin, Ireland; 2 Department of Psychology, Maynooth University, Maynooth, County Kildare

**Keywords:** Coronavirus, COVID-19, epidemic, infectious disease outbreak, pandemic, rapid review, youth mental health

## Abstract

**Objectives:**

In March 2020, the World Health Organization (WHO) officially declared the spread of coronavirus disease 2019 (COVID-19) as a pandemic. Adolescence and early adulthood are peak times for the onset of mental health difficulties. Exposure to a pandemic during this vulnerable developmental period places young people at significant risk of negative psychological experiences. The objective of this research was to summarise existing evidence on the potential impact of a pandemic on the mental health of 12–25 year olds.

**Methods:**

A rapid review of the published peer-reviewed literature, published between 1985 and 2020, using PsycINFO (Proquest) and Medline (Proquest) was conducted. Narrative synthesis was used across studies to identify key themes and concepts.

**Results:**

This review found 3,359 papers, which was reduced to 12 papers for data extraction. Results regarding the prevalence of psychological difficulties in youth were mixed, with some studies finding this group experience heightened distress during an infectious disease outbreak, and others finding no age differences or higher distress among adults. Gender, coping, self-reported physical health and adoption of precautionary measures appear to play a role in moderating the psychological impact of an infectious disease outbreak. Most studies were conducted after the peak of an epidemic/pandemic or in the recovery period.

**Conclusions:**

More longitudinal research with young people, particularly adolescents in the general population, before and during the early stages of an infectious disease outbreak is needed to obtain a clear understanding of how best to support young people during these events.

## Introduction

A pandemic is characterised by the simultaneous worldwide spread of a novel infectious disease and typically causes widespread economic, social and political disruption (Doshi, 2011; Kelly, 2011). Although infrequent, some evidence suggests that globalisation has increased the likelihood of their occurrence (Madhav e*t al*. 2017). Individuals affected by an infectious disease outbreak, such as a pandemic, often experience increased anxiety, particularly around contracting the illness, a higher incidence of mental health difficulties and heightened feelings of helplessness and stigma (Hall *et al*. 2008; Douglas *et al*. 2009; Rubin *et al*. 2010; Sim *et al*. 2010; Kelly, 2020). Mitigating the impact of a pandemic typically requires a large-scale, coordinated public health response [World Health Organization (WHO), 2017]. Risk-based measures including social/physical distancing, travel or movement restrictions, school/business closures and enforced quarantine to slow the spread of the disease and lessen its impact on the health system are often taken (WHO, 2018a). Thus, the negative psychological impact of a pandemic can be compounded by the public health measures introduced to contain the virus (Van Bortel *et al*. 2016; Holmes *et al*. 2020). Indeed, a series of recent reviews on the effects of quarantine and social isolation indicate they can lead to prolonged mental health difficulties (Brooks *et al*. 2020; Hossain *et al*. 2020; Loades *et al.* 2020).

Young people between 10 and 24 years of age account for almost a quarter of the total global population (Gupta, 2014; The World Bank, 2018). Adolescence and early adulthood are critical periods of development, which can shape the likelihood, severity and course of mental health problems (Kessler *et al*. 2007; Kessler *et al*. 2012). Many young people are attending school or university, which are among the first institutions to close as part of infection prevention measures, leaving them isolated from their peer groups as well as primary help-seeking and support facilities (Fegert & Schuzle, in press; Stevenson *et al*. 2009; Van *et al.* 2010; WHO, 2017; Holmes *et al.* 2020; Kelly, 2020; WHO, 2017). Additionally, family distress is often high during a pandemic and young people may find themselves coping with feelings of distress and anxiety in the face of compromised support structures (Douglas *et al*. 2009).

On 11th March 2020, the WHO officially declared the spread of the coronavirus disease 2019 (COVID-19) as a pandemic. At the time of writing, there were over 4.3 million confirmed cases of COVID-19 across 188 countries/regions, with over 290 000 associated deaths (John Hopkins University, 2020). Resulting public health responses have included widespread restrictions on social activity and closures of public spaces, schools and non-essential businesses (Bedford *et al.* 2020; Sohrabi *et al.* 2020). Emerging research on the COVID-19 outbreak indicates that over half (53.8%) of individuals rate the psychological impact of the pandemic as moderate to severe (Wang *et al*. 2020). Another nationwide study with 52 730 respondents across 36 provinces in China, the country at the centre of the COVID-19 outbreak, found that over one-third (35%) of individuals reported symptoms of psychological distress (Qiu *et al*. 2020). Others have suggested that individuals with confirmed and suspected cases of COVID-19 may experience fear of severe disease consequences and the contagion, and have increased risk of suicide (Li *et al.* 2020, Lin, 2020).

Exposure to the COVID-19 pandemic during a vulnerable developmental stage places young people at a greater risk of the negative psychological impacts of such an event (Holmes *et al*. 2020). The objective of this rapid review was to summarise the information available about the potential impact of a pandemic on the mental health of young people aged 12–25 years. This age range was selected as it reflects international trends in current service provision for young people, research in this area (Hetrick e*t al*. 2017) and the WHO definition of youth (United Nations [UN], 2013).

## Method

### Rapid review methods

A rapid review was conducted to capture relevant studies related to the research question. Rapid reviews condense the systematic review process to provide robust evidence-informed decisions in a cost-effective manner. This method is particularly appropriate when information and evidence is required quickly and in times of crisis (Tricco *et al*. 2017). The review was documented using the Preferred Reporting items for Systematic Reviews and Meta-Analysis (PRISMA) guidelines. The protocol was registered with PROSPERO (CRD42020177796).

This review included all types of studies that explored how the mental health (outcome) of young people aged 12–25 years (population) could be affected by an exposure to a pandemic (exposure). This review was limited to studies relating to exposure to an infectious disease outbreak classified as either an ‘epidemic’ or ‘pandemic’, as these terms are often used interchangeably in the literature. This includes infectious disease outbreaks such as COVID-19, H1N1/swine flu, severe acute respiratory syndrome (SARS), Middle East respiratory syndrome (MERS), Ebola and HIV/AIDS. Studies examining treatments or risk factors for infectious diseases or exclusively focusing on populations such as healthcare workers were excluded. The focus was on studies where the majority of participants were aged 12–25 years, or where a sub-group of participants was clearly identified as being within this age range. For the purposes of the review, the WHO (2018b, para. 2) definition of mental health as ‘a state of well-being in which every individual realises his or her own potential, can cope with the normal stresses of life, can work productively and fruitfully and is able to make a contribution to his or her own community’. There was no geographical restriction on papers. The search was restricted to English, peer-reviewed abstracts and titles in PsycINFO (Proquest) and Medline (Proquest) from January 1985 to March 2020. Further details on the rapid review method and our search and selection strategy are provided in Appendix A.

### Consultation with experts

In keeping with recommendations from the Cochrane Rapid Reviews Methods Group (Garritty *et al*. 2020), the research team sought input from *N* = 30 youth mental health professionals working in a large youth mental health organisation based in Ireland in refining our research question. Respondents provided positive feedback to the research team and highlighted the potential application of the findings in the field.

### Data synthesis

A quantitative synthesis proved to be inappropriate due to the heterogeneity of study designs, contexts and outcomes in the literature. Thus, a narrative synthesis across studies was used to identify key themes and concepts. Narrative synthesis refers to an approach to that relies chiefly on the use of words and text to summarise and explain the findings of the synthesis (Popay *et al*. 2006). First, the characteristics and findings of individual studies were tabulated, eligible studies were read and re-read independently by members of the research team and initial themes were generated (i.e. preliminary synthesis). As per Popay *et al’s*. (2006) guidelines on narrative synthesis, variations in outcomes, study design, populations and content were noted, and relationships within and across studies were documented. Themes were then discussed and reviewed by the whole research group and against the full data set. As themes emerged from a review of the primary data, this remains an inductive approach (Atkins *et al*. 2008).

## Results

### Search results

**Fig. 1.**
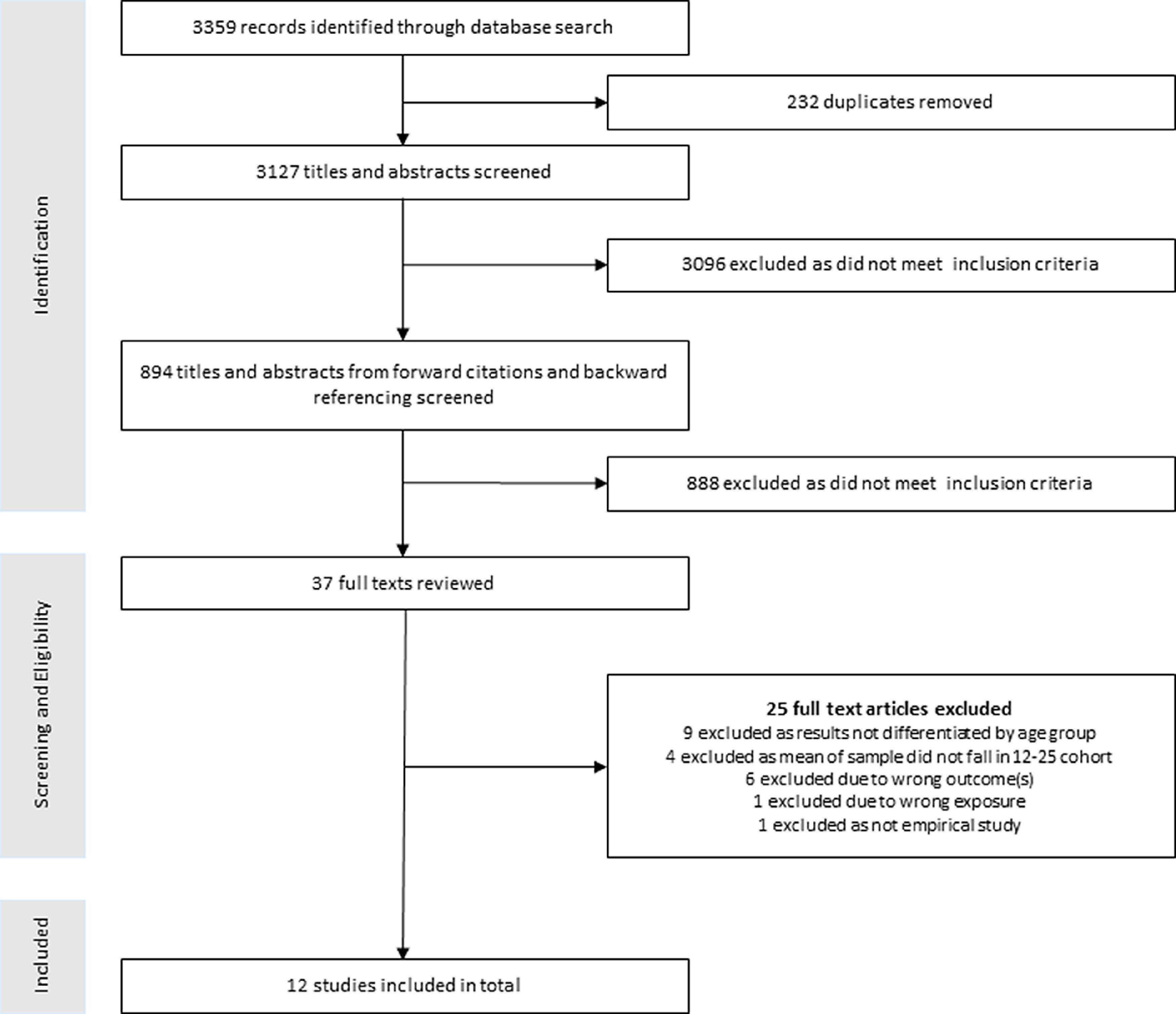
Rapid review of peer-reviewed publications in the scientific literature: study selection.

Initial searches yielded 3,359 search results, which was reduced to 3,127 after duplicates were removed. The screening review process is illustrated in Fig. 1. Initially, two members of the research team (MT, BMcK) reviewed the titles and abstracts of approximately half the papers each to make an initial assessment of relevance. Similar to Brown *et al*. (2020), following the initial search, papers that related to the HIV/AIDS pandemic were excluded as the mode of transmission is different (i.e. it is not an airborne transmission). A random sample of 10% of titles/abstracts were examined by two additional reviewers (AB, AOR). Discrepancies (*N* = 17) were resolved through discussion. After this initial screening, 3,096 papers failed to meet the inclusion criteria, leaving 31 papers for full screening by two members of the research team (MT, BMcK). Forward and backward reference checking of key articles yielded a further six studies, leaving 37 papers for full screening.

After full screening, a further 25 papers failed to meet the inclusion criteria, leaving a final list of 12 papers for data extraction. Data were extracted onto a template by two researchers (MT, BMcK). Variables to be extracted comprised of the following: country of origin, study design, aims, method, participant characteristics, method of data analysis and key findings. This information was stored on a Microsoft Excel database. The remaining papers were examined using the appropriate Joanna Briggs critical appraisal checklist (Aromataris *et al*. 2015). These checklists have been widely used in rapid reviews and allow for the quick evaluation of study quality.

### Study characteristics and quality

The studies included seven prevalence studies, three cross-sectional studies, one longitudinal study and one case-control study (see Tables 1 through 3). Most (5/12) of the studies were undertaken in China, followed by Taiwan (2/12) and Hong Kong (2/12), with one each from Canada, Sweden and Saudi Arabia. Half of the studies (6/12) included 12–25-year-olds as part of larger studies with members of the public, 4/12 were conducted with university students, one with medical students and one with children and young people who had developed narcolepsy after receiving the H1N1 vaccine. The majority of studies were related to the SARS outbreak (9/12), and one each to H1N1, MERS and COVID-19, respectively. Sample sizes were mostly modest, and varied from *N* = 38 (Szakács *et al*. 2015) to *N* = 4,481 (Leung *et al*. 2005). Two-thirds of the studies were rated as being of moderate quality (8/12), while one-third were rated as high quality (4/12).

**Table 1.** Cross-sectional studies

Citation	Sampling strategy	Participants	Region	Exposure	Aim and study design	Outcome measures	Main findings 12–25 age group	Joanna Briggs quality appraisal
Mihashi *et al*. (2009)	Cluster sampling from two college campuses; Workers, university faculty and family members in campus ‘A’, and students located in campus ‘B’	*N* = 187 32% female; 54% male; <1% not specified Mean 26.3 ± 8 years Results disaggregated by age: sub-group <23 years	Beijing, China	SARS	Investigated strategies for broad mass isolation during outbreaks of infectious diseases during the SARS outbreak recovery period Cross-sectional study using directly delivered paper surveys	Psychological disorders (GHQ-30; Goldberg & Williams, 1988)	No significant differences in psychological disorders between age groups (*OR* = .8) 24% of those under 23 years reported psychological symptoms indicative of a psychological disorder	**Medium**
Szakács *et al*. (2015)	Purposive sampling from population-based study	*N* = 38 55% female; 45% male 5–25 years	Sweden	H1N1 influenza	Evaluated psychiatric comorbidity and the cognitive profile of children and adolescents with narcolepsy in western Sweden and the relationship of these problems to H1N1 vaccination Cross-sectional study using a test battery of semi-structured interviews	Psychiatric comorbidity [suite of measures to assess meeting of ICD-10, WHO, 1993 and DSM-IV criteria; American Psychiatric Association (APA), 2000] Cognitive Profile (Age-appropriate Wechsler Intelligence test battery)	Rates of psychiatric difficulties were higher in post-H1N1 vaccinated participants than those with non-post-H1N1 narcolepsy (*OR* = 4.6^a^) Major depression in post-H1N1 vaccinated participants was three times higher than the prevalence in the general adolescent population^b^	Medium
Xie *et al*. (2011)	Cluster sampling of residents in infected and non-infected areas and convenience sampling	*N* = 647 35% female; 65% male; <1% not specified Mean 23.8 years	China	SARS	Examined effect of SARS crisis on levels of distress during the SARS epidemic in China Cross-sectional study using online surveys	Anxiety (STAI-Form Y, Chinese version; Spielberger *et al*. 1983) Imitative behaviour (author designed measure)	Anxiety levels were higher in non-infected areas than quarantined or nearby quarantined areas^b^ Anxiety was predicted directly by SARS-related vigilance, less willingness to volunteer, perceived impact, lower levels of perceived knowledge and pessimism^b^	Medium

SARS , severe acute respiratory syndrome; H1N1, influenza A sub-type H1N1; GHQ-30 , 30-item General Health Questionnaire; ICD-10, 10th revision of the International Statistical Classification of Diseases and Related Health Problems; DSM-IV, Diagnostic and Statistical Manual of Mental Disorders; STAI-Form Y , State-Trait Anxiety Inventory (Form Y); OR, odds ratio.

Joanna Briggs Quality appraisal rating is based on percentage of criteria met for appropriate study type, for the purposes of this study high ≥ 70%, medium = 30–70% and low ≤ 30%.

^a^ Effect size calculated from available data.

^b^ Insufficient data available to calculate effect size.

**Table 2.** Prevalence studies

Citation	Sampling strategy	Participants	Region	Exposure	Aim and study design	Outcome measures	Main findings 12–25 age cohort	Joanna Briggs quality appraisal
Al-Rabiaah *et al*. (2020)	Random sampling of university medical students	*N* = 174 40% female; 60% male Mean 21.6 ± 1 years	Saudi Arabia	MERS-CoV	Examined the impact of MERS on medical students’ perception and determinants of their psychological distress during outbreak Cross-sectional study using online questionnaires	Stress levels (1–10 rating) Anxiety levels (GAD-7; Spitzer *et al*. 2007)	77% reported minimal, 18.4% reported mild and 4.6% reported moderate levels of anxiety ^•^ Females had significantly higher stress levels than males (*d* = .4^a^) Perceived social avoidance score (*β* = .2), improved hygiene habits (*β* = .3) significantly predicted higher stress levels Knowledge of MERS-CoV (*β* = .1) agreeing with public fear (*β* = .1) and number of resources accessed (*β* = .1) did not significantly predict stress levels	High
Bergeron, & Sanchez (2005)	Random sampling of university students	*N* = 300 73% female 18–23 years Mean 21.1 ± 5 years	Canada	SARS	Examined preferences and use of various types of mass communication media, anxiety levels of acquiring the infection and general knowledge of SARS in university students after the 2003 SARS outbreak Cross-sectional study using online and paper questionnaires	Access and use of mass communication media Level of anxiety (unspecified 7-item scale)	43% of student reported high levels of anxiety Anxiety was not significantly associated with the use intensity of any type of media^b^ Gender (being female) and area of residence (in the greater Toronto area) was significantly associated with high levels of anxiety^b^	Medium
Gan *et al*. (2004)	Random sampling of university students	*N* = 93 58% female; 42% male Mean 22.1 ± 3 years	Beijing, China	SARS	Examined the coping flexibility of university students in response to SARS-related and daily life stressful events Cross-sectional study	Coping flexibility (CFQ; Cheng, 2001) Daily life stressful events (items from ULES; Wang & Gan, 1994, ICSRLE; Kohn *et al.* 1990 & SRRS; Reale, 1987) SARS-related stressful events (author designed scale)	Perceived controllability was significantly lower for SARS-related stress compared to daily life stress (*d* = 1.2^a^) No significant difference in perceived effectiveness of coping behaviour between SARS-related stress and daily life stress (*d = *.2^a^) Coping flexibility was lower for SARS-related stress than daily life stress^b^	Medium
Lau *et al*. (2005)	Random sampling of general population in Hong Kong from phone directories	Survey 1: *N* = 863 50% female; 50% male 18–60 years Survey 2: *N* = 818 50% female; 50% male 18–60 years Results disaggregated by age: sub-group 18–29	Hong Kong, China	SARS	Examined perceptions and mental health effects of SARS on the general population in Hong Kong during the end phase of the epidemic Cross-sectional study using two telephone surveys	Survey 1: SARS-related perceptions Survey 2: Psychological effects (self-reported) Psychological effects (IES, Chinese version; Horowitz *et al*. 1979) Mental health (2 * SF-36 sub-scales; Ware, 1992)	No significant difference in psychological effects or quality of life between age groups^b^ 18–24-year-olds had significantly lower odds of perceiving an overall effect on mental health than those aged 25–34 (*OR* = .6^a^) or 50+ (*OR* = .5^a^). No significant difference in having trouble falling/staying asleep, having a psychosomatic response or perceived need for a psychiatrist or psychologist between age groups^b^	High
Main *et al*. (2011)	Cluster sampling of university students from two public universities.	*N* = 381 43% male 17–24 years Mean 20.2 ± 1 years	Beijing, China	SARS	Examined the main effects and interactions between SARS-related stressors and coping strategies and Chinese college students’ psychological adjustment at the end of the 2003 Beijing SARS epidemic Cross-sectional study using paper questionnaires	Psychological symptoms (4 * SCL-90 sub-scales, Chinese version; Derogatis, 1977) Life satisfaction (Life Satisfaction Scale; Diener *et al*. 1985) Perceived general health (self-rated)	No significant gender difference in psychological symptoms (*d* = .01^a^). Females reported significantly higher levels of life satisfaction than males (*d* = .3^a^) Experience of SARS-related stressors was positively associated with psychological symptoms (*β* = .1) Interaction between SARS-related stressors and coping predicted perceived general health (*β* = .1) but not psychological symptoms (*β* = −.1) or life satisfaction (*β* = −.02)	Medium
Peng *et al*. (2010)	Stratified random sampling of general population based on geographic area	*N* = 1,278 50% female; 50% male 18–89 years Mean 41.6 ± 17 years Results disaggregated by age: sub-group 18–29	Taiwan	SARS	Explored post-crisis psychological distress in Taiwan residents after the SARS epidemic Cross-sectional study using computer-assisted telephone interview systems	Change in perception of life (pessimism) Psychological distress (BSRS-5; Lung & Lee, 2008)	18–29-year-olds had significantly greater odds of having severe psychological distress than those aged 50+ (*OR* = 2.0–2.5^a^)18–29-year-olds had significantly lower odds of feeling more pessimistic after the resolution of the SARS crisis than those aged 60+ (*OR* = .4^a^)	High
Wang *et al*. (2020)	Snowball sampling of general population	*N* = 1,210 67% female; 33% male 12–59 years Results disaggregated by age: sub-groups 12–21; 21–31 years	China	COVID-19	Examined levels of psychological impact, anxiety, depression and stress on the general public during the initial stage of the COVID-19 outbreak Cross-sectional study using online questionnaires	Psychological impact (IES-R; Weiss, 2007) Mental health status: stress, anxiety and depression (DASS-21; Henry & Crawford, 2005)	Student status was significantly associated with higher psychological impact (*β* = .2), stress (*β* = .1), anxiety (*β* = .2) and depression (*β* = .1) compared to those who were employed No significant difference in psychological impact, stress, anxiety or depression between age groups (*β* = .1-.2)	Medium

MERS-CoV, Middle East respiratory syndrome coronavirus; SARS, severe acute respiratory syndrome; COVID-19, coronavirus disease 2019; GAD-7, 7-item General Anxiety Disorder scale; CFQ, Coping Flexibility Questionnaire; ULES, University Life Event Scale; ICSRLE, Inventory of College Students Recent Life Experiences; SRRS, Social Readjustment Rating Scale; IES-R, Impact of Event Scale – Revised; SF-36*, 36-item Short Form Health Survey, 2 sub-scales used: mental health and vitality/quality of life; SCL-90*, 90-item symptom checklist, 4 sub-scales used: somatisation, obsessive-compulsive, depressive and phobic/anxiety symptoms; BSRS-5, Brief Symptom Rating Scale; DASS-21, The Depression, Anxiety and Stress Scale; *d*, standardised mean difference*; β*, standardised regression coefficient; OR , odds ratio.

Joanna Briggs Quality appraisal rating is based on percentage of criteria met for appropriate study type, for the purposes of this study high  ≥ 70%, medium = 30–70%, and low ≤ 30%.

^a^ Effect size calculated from available data.

^b^ Insufficient data available to calculate effect size.

**Table 3.** Other studies

Citation	Sampling strategy	Participants	Region	Exposure	Aim and study design	Outcome measures	Main findings 12–25 age cohort	Joanna Briggs quality appraisal
Leung *et al*. (2005)	Random sampling of general population (18+)	*N* = 4,481 Gender: NR 18–65+ years Results disaggregated by age: sub-group 18–24 years	Hong Kong, China	SARS	Examined psychological and behavioural responses to the SARS outbreak over time Longitudinal and repeated cross-sectional design involving 6 population based surveys conducted using random digit dialling	Anxiety symptoms (STAI; Spielberger *et al*. 1983) Adoptions of precautionary measures.	Anxiety symptoms for 18–24-year-olds decreased over time after infectious disease outbreak^b^ 18–24-year-olds had lower anxiety symptoms than 25–44-year-olds at the peak of the outbreak but not at later time points^b^	High
Ko *et al*. (2006)	Random sampling of general population (15+)	*N* = 1,473 51% female; 49% male 15–50+ years Results disaggregated by age: sub-group 15–30 years	Taiwan	SARS	Examined the psychological state of those impacted *v.* those not impacted by SARS following its outbreak Case control study using telephone interviewing	Depression level (TDQ; Lee *et al*. 2020) Self-Perceived Health Questionnaire Neighbourhood Relationship Questionnaire	No significant difference in depression level between age groups in those impacted by SARS (*d*<.01^a^)	Medium

SARS, severe acute respiratory syndrome; STAI, State-Trait Anxiety Inventory; TDQ, Taiwanese Depression Questionnaire; *d*, standardised mean difference.

Joanna Briggs Quality appraisal rating is based on percentage of criteria met for appropriate study type, for the purposes of this study high ≥ 70%, medium = 30–70% and low ≤ 30%.

^a^ Effect size calculated from available data.

^b^ Insufficient data available to calculate effect size.

### Narrative synthesis

Three major themes emerged from the narrative synthesis: prevalence of psychological difficulties among youth, factors moderating psychological difficulties and aspects of infectious disease outbreak causing distress.

### Prevalence of psychological difficulties

There was some variation in findings regarding the prevalence of psychological difficulties among youth affected by an infectious disease outbreak. Four studies reported high anxiety or distress among young people recruited from the general population, university and health services during or following an outbreak (Bergeron & Sanchez, 2005; Peng *et al*. 2010; Main *et al*. 2011; Szakács *et al*. 2015), while another found student status was predictive of greater psychological distress (Wang *et al*. 2020). However, other studies found older age groups reported higher levels of distress (25–44 -year-olds; Leung *et al*. 2005), perceived the pandemic had a greater impact on their mental health (25–34-year-olds and those aged 50+; Lau *et al*. 2005) or were more pessimistic (those aged 60+; Peng *et al*. 2005). Two studies found no age differences (Ko *et al*. 2006; Mihashi *et al*. 2009). Additionally, one study with university medicine students found that participants generally reported low levels of anxiety (Al-Rabiaah *et al*. 2020). A final study found young people in epidemic areas, which were described as ‘the eye of the storm’, were less anxious than those in non-epidemic areas, although the sample size was small (Xie *et al*. 2011). As shown in Tables 1 through 3, there was significant variation in how mental health outcomes were measured, with some studies using author-designed measures, and others using standardised measures of anxiety [e.g. Depression Anxiety Stress Scale (DASS-21), Henry & Crawford, 2005; State-Trait Anxiety Inventory (STAI), Spielberger *et al*. 1983; General Anxiety Disorder (GAD-7); Spitzer *et al*. 2007], psychological disorder [e.g. General Health Questionnaire (GHQ-30), Goldberg & Williams, 1988] or distress [e.g. Brief Symptom Rating Scale (BSRS-5), Lung & Lee, 2008; Impact of Event Scale (IES-R), Weiss, 2007].

### Factors moderating psychological difficulties

Gender was only examined in three studies among the target age group. Two studies found female university students reported significantly higher levels of psychological distress than their male peers (Bergeron & Sanchez, 2005; Al-Rabiaah *et al*. 2020). The results from a third study indicated male and female university students were equally affected by the SARS epidemic, although female students reported higher life satisfaction (Main *et al*. 2011). This study also found that, in general, all types of coping (i.e. active coping, avoidant coping and support focused coping) served as a buffer against the negative impact of stressors on perceived health, although female students reported less passive coping than their male peers. Additionally, Gan *et al*. (2004) reported that Chinese university students used less flexible coping strategies when dealing with SARS-related stress in comparison to daily life stresses, mirroring the coping reactions of individuals with depression.

It was notable that few studies asked participants to provide information on their physical health, given many individuals often experience physical illness during infectious disease outbreaks. Although four studies (Ko *et al*. 2006; Mihashi *et al*. 2009; Main *et al*. 2011; Wang *et al*. 2020) found self-reported health status was significantly associated with psychological difficulties, only one of these presented results for the target age group. Here, the authors observed a significant moderate positive correlation between psychological symptoms and general health among university students (Main *et al*. 2011). Another study looked at SARS-related vigilance among the general population, and found participants consistently thinking about whether or not they had contracted SARS was linked with higher levels of anxiety (Xie *et al.* 2011). Finally, two studies examined the relationship between the adoption of precautionary measures and psychological distress among young people. Results from both studies indicated that the adoption of precautionary measures such as avoiding others and greater change in hygiene habits was significantly associated with higher levels of anxiety/stress (Xie *et al*. 2011; Al-Rabiaah *et al.* 2020).

### Aspects of infectious disease outbreak causing distress

The timing of data collection varied across studies. With the exception of one longitudinal study, which comprised multiple phases of data collection with members of the general population during and after a SARS outbreak (Leung *et al.* 2005), most studies were conducted when an outbreak had been controlled or after the resolution of this event. While the longitudinal study did show an overall decrease in anxiety in a population from the peak of an epidemic to post-epidemic, and this trend was observed among 18–24-year-olds, results were not significant for this age group (Leung *et al*. 2005)

In addition to studies looking at general difficulties during a pandemic, others focused on events linked to an infectious disease outbreak. A small number focused on social isolation or quarantine among youth. As noted earlier, in one study, the authors found that participants in epidemic areas were generally less anxious than those in non-epidemic areas (Xie *et al*. 2011). A second study found exposure to more SARS stressors, including having to cancel planned activities, predicted psychological difficulties among university students (Main *et al.* 2011). Other studies pointed to a relationship between being quarantined or living with restrictions and psychological well-being, but results were not disaggregated by age (Ko *et al*. 2006; Mihashi *et al*. 2009; Peng *et al.* 2010).

Finally, there were two studies that looked at specific factors associated with a pandemic. One study examined media use and its link to mental health among university students, but found anxiety levels were not associated with the use intensity of any type of media (Bergeron & Sanchez, 2005). Another study examined psychological difficulties among children and adolescents who had developed narcolepsy after receiving a vaccine for H1N1, and found higher prevalence of psychiatric disorders among this group compared to those who had developed narcolepsy due to other reasons (Szakács *et al*. 2015).

## Discussion

The purpose of this rapid review was to synthesise and describe the available evidence on the potential impact of a pandemic on young people’s mental health. There is generally consensus in the literature that rates of anxiety and depression across countries among adolescents and young adults have increased (Collishaw, 2015; Mojtabai *et al.* 2016; Dooley *et al*. 2019; Patalay & Gage, 2019). How young people’s mental health is affected by an infectious disease outbreak and the public health measures to control such an outbreak is unclear. This review revealed some studies in this area point to heightened vulnerability among youth, others suggest adults are more affected – possibly due to increased physical health risks (Mackay & Arden, 2015) – and a small number report no age differences. The research almost consistently indicates females report higher levels of distress, which mirrors the broader literature with this age group (Dooley *et al.* 2019; Patalay & Gage, 2019; Wiens *et al*. 2020).

The disparity observed may be somewhat explained by the different instruments used to assess mental health/psychological well-being. While several studies used standardised questionnaires to examine a particular aspect of distress, others used one-item author-designed measures. It is also worth noting that most (75%) of the studies were conducted in Chinese or other Eastern cultures, where a number of recent infectious disease outbreaks have occurred. Eastern and western cultures typically respond differently to negative emotions (Furlong & Finnie, in press). Individuals from collective cultures tend to report more somatic symptoms than psychological symptoms (Ryder *et al.* 2008), and coping strategies are also likely to vary depending on culture (Chun *et al.* 2006), pointing to the need to consider the larger social and cultural context in addition to the situational context of a pandemic (Wong *et al.* 2006). Indeed, two studies point to the importance of adaptive coping styles in responding to adversity during an infectious disease outbreak. Maladaptive coping is a risk factor for the development of psychological difficulties after a pandemic or natural disaster (Coetzee & Spangenberg, 2003; Naushad *et al.* 2019).

It was surprising that the majority of the research included in this review was conducted in the latter stages or after an infectious disease outbreak. None of the studies reviewed included data collection points prior to and after an infectious disease outbreak, meaning the ability to infer changes in youth mental health as a direct result of the outbreak is significantly limited. There is a real need to conduct more longitudinal research, particularly prior to and during the peak stages of an infectious disease outbreak, when young people are most likely to be affected by public health measures or feel particularly anxious about their physical health. Although previous research has established a link between the impact of social isolation, quarantine and restricted movements and distress (Brooks *et al.* 2020; Hossain *et al.* 2020; Loades *et al.* 2020), we could not draw firm conclusions from this review on how young people are affected by such measures during a pandemic. The COVID-19 pandemic is much more widespread than the other infectious disease outbreaks described in many of the papers included in this review, and the long-term economic effects are likely to be more significant, particularly for young people [Oswald & Powdthavee, 2020; Institute for Fiscal Studies (ISF), 2020]. Previous research has indicated youth and parent unemployment can have a significant psychological impact on young people (Fergusson *et al.* 2001; Virtanen *et al.* 2016). Conversely, the successful recovery of national economies appears to crucially depend on the mental health of the population (WHO, 2011).

It is worth noting that most of the studies included in this review used convenience, non-representative samples. Although seven studies reported some element of random sampling, the samples were typically restricted to a particular geographic or educational setting. Only two studies reported random sampling based on a specified sampling frame, thus limiting the ability to make accurate inferences about prevalence. The studies were also typically comprised of university students, and none were conducted solely with 12–25-year-olds. Only four studies included young people under the age of 18, meaning we are limited in our ability to make inferences about prevalence particularly in terms of how adolescents may be affected by a pandemic. Additional research with young people with pre-existing mental health difficulties or those experiencing challenges with regards to their personal, family or social circumstances are warranted, as this group may be disproportionally affected by the medium- and long-term social effects of COVID-19, and resource allocation for youth mental health services is generally insufficient (Brown *et al.* 2020; Furlong & Finnie, in press; Li *et al.* 2020). The voice of young people is also notably absent from the literature on this topic. Patient and public involvement is critical to understanding people’s lived experiences, yet the methods adopted in the existing body of research do not actively promote youth voice. It is important any research with young people is ethically robust and researchers view COVID-19 mental health research as a sensitive topic, where attention is paid to the safeguards needed to protect the well-being of participants (Townsend *et al.* 2020). This is particularly salient for research with young people under the age of 18, where legal and developmental considerations limit their capacity to consent independently and parental support may be required (Hiriscau *et al.* 2016).

### Strengths/limitations of study

This review is the first to focus on the mental health impacts of a pandemic on the 12–25-year-old cohort, which is a target age group for a growing number of youth mental health services internationally (Hetrick *et al*. 2017). Incorporating a consultation with mental health professionals to refine the research question, collaborating with a young person as an author on the rapid review team, adopting a systematic process of study selection and rigorous synthesis methods are all key strengths of the review.

However, the review conclusions are ultimately limited by the quality of the primary studies reviewed. Although all of the studies identified in this review were rated as moderate or high in terms of quality, convenience sampling, an absence of strategies to deal with confounding factors, variation in measures used to assess the primary outcome (mental health) and heterogeneity of outcome measures in the studies identified are all limitations of the review. As noted above, there is also a notable absence of studies with adolescents or incorporating youth perspectives in the reviewed studies. In addition, the predominance of cross-sectional data gathered in Eastern cultures in the period before/after a pandemic limits our ability to draw conclusions about the immediate or subsequent long-term impacts of a pandemic on youth mental health. Further, slightly more than half of the studies included respondents outside the 12–25-year-old age group, most of which contained only limited, albeit valuable, information that was disaggregated for this age cohort. Additionally, in order to quickly collate the evidence available, this review employed single-reviewer screening with 10% verification by a second reviewer, which is common in rapid reviews (Abou-Setta *et al*. 2016). Finally, the review focused on peer-reviewed, English language publications and may not have identified all related existing and emerging published/unpublished publications related to pandemics.

### Recommendations for practice

Few studies have considered the collective impact of biological, social and psychological risk and protective factors on youth mental health, meaning our ability to make recommendations about how to effectively intervene and impact on young people’s mental health during a pandemic is limited. However, some considerations for practice and policy are evident. First, this review highlights mental health should be considered as part of a holistic response to the COVID-19 outbreak. Second, while cultural context must be considered, there are indicators that adaptive coping styles can support young people’s capacity to navigate through an uncontrollable event such as a pandemic, pointing to an area of intervention for mental health service providers. Psychological interventions incorporating cognitive behavioural therapy or problem-solving therapy may be valuable, and could be delivered online. Delivery of online services and the integration of e-therapy tools have begun in many countries as a result of the COVID-19 outbreak (Wind *et al.* 2020). Community-based workshops or health promotion campaigns could also focus on the promotion of adaptive coping styles. Finally, this review highlights the need to take factors such as age and gender into account when delivering mental health campaigns to support populations in the aftermath of COVID-19.

## Conclusion

During an infectious disease outbreak, the focus of research and action is often on the medical and public health communities, where it has typically (rightly) been on the identification of the responsible agent, clinical presentation and treatment of the disease (Leung *et al.* 2005). However, it is important to pay attention to the ways a pandemic can impact on mental health. To the best of the author’s knowledge, this is the first time the evidence on young people’s mental health during a pandemic has been synthesised. On the basis of the review, we are unable to determine the extent by which – if at all – young people’s mental health is affected by a pandemic, what factors may mitigate the impact of a pandemic on mental health, and how culture/context could affect this impact. The review highlights there has been minimal consideration of how this group can been affected by a pandemic, and points to an urgent need for more research on this area, particularly with adolescents. The COVID-19 crisis has been described as ‘unprecedented, prolonged and unpredictable’ (Pūras, 2020) and the impact on youth well-being needs to be considered as a priority.

## Appendix A

Rapid Review ProtocolSearch Strategy Search strategies were developed using both keywords and MeSH terms. The search strategies were modified for each included database (PsycInfo and Medline). The * is a wildcard to search for terms that begin with the given string. Keywords and MeSH terms were searched for in title and abstract. Both databases were searched for papers between January 1985 to March 2020. Searches were restricted to English language papers only, in peer-reviewed journals. PsycInfo Search Strategy: PsycInfo was searched using the ProQuest interface on 06.04.2020 (temporal coverage from 1887- present). Search terms included: (Ab(“young people” OR youth OR adolescen* OR “young adult” OR teen* OR child* OR youth OR “young person*” OR juvenile OR minors OR “emerging adult”) OR (MJMAINSUBJECT.EXACT(“Early Adolescence”) OR MJMAINSUBJECT.EXACT(“Emerging Adulthood”))) AND (ab(pandemic OR epidemic OR COVID-19 OR HIV/AIDS OR h1n1 OR MERS OR SARS OR ebola OR quarantin* OR “self isolation”) OR (MJMAINSUBJECT.EXACT(“Pandemics”) OR MJMAINSUBJECT.EXACT(“Epidemics”) OR MAINSUBJECT.EXACT(“HIV”) OR MAINSUBJECT.EXACT(“AIDS”) OR MAINSUBJECT.EXACT(“Swine Influenza”))) AND (ab(“mental health” OR “quality of life” OR “happiness with life” OR “life satisfaction” OR resilien* OR “depress*” OR “anxi*” OR “PTSD” OR “posttraumatic stress” OR loss OR bereavement OR grief OR psychological OR psychiatric OR insomnia OR psychosocial OR “key wellness” OR wellbeing) OR (MAINSUBJECT.EXACT(“Grief”) OR MJMAINSUBJECT.EXACT(“Depression (Emotion)”) OR MAINSUBJECT.EXACT(“Mental Health”) OR MAINSUBJECT.EXACT(“Complex PTSD”) OR MAINSUBJECT.EXACT(“Major Depression”) OR MAINSUBJECT.EXACT(“Anxiety Disorders”) OR MJMAINSUBJECT.EXACT(“Resilience (Psychological)”))) AND (la.exact(“ENG”) AND PEER(yes)) Medline Search Strategy: Medline was searched using the ProQuest interface on 06.04.2020 (coverage from 1946 – present). Search terms included: ((ab(“young people” OR youth OR adolescen* OR “young adult” OR teen* OR child* OR youth OR “young person*” OR juvenile OR minors OR “emerging adult”) OR (MESH.EXACT(“Adolescent”) OR MESH.EXACT(“Young Adult”) OR MESH.EXACT(“Child”))) AND (ab(pandemic OR epidemic OR COVID-19 OR HIV/AIDS OR h1n1 OR MERS OR SARS OR ebola OR quarantin* OR “self isolation”) OR (MESH.EXACT(“HIV”) OR MESH.EXACT(“Epidemics”) OR MESH.EXACT(“Influenza A Virus, H1N1 Subtype”) OR MESH.EXACT(“Pandemics”))) AND (ab(“mental health” OR “quality of life” OR “happiness with life” OR “life satisfaction” OR resilien* OR “depress*” OR “anxi*” OR “PTSD” OR “posttraumatic stress” OR loss OR bereavement OR grief OR psychological OR psychiatric OR insomnia OR psychosocial OR “key wellness” OR wellbeing) OR (MESH.EXACT(“Mental Health”) OR MESH.EXACT(“Anxiety Disorders”) OR MESH.EXACT(“Bereavement”) OR MESH.EXACT(“Resilience, Psychological”) OR MESH.EXACT(“Grief”) OR MESH.EXACT(“Stress Disorders, Post-Traumatic”)))) AND (la.exact(“ENG”) AND pd(19850101-20201231) AND PEER(yes)) Forward citations were searched using Google Scholar on 23.04.2020 and included/excluded for full-review based on the same inclusion/exclusion criteria as initial searches. Where full-texts could not be accessed following initial full-text screening, relevant authors were contacted.
